# Case of Nontraumatic Rectus Sheath Hematoma from Muscle Training Mimicking Acute Abdomen

**DOI:** 10.1155/2019/3158969

**Published:** 2019-05-23

**Authors:** Yukino Ariyoshi, Hiromichi Naito, Hiromi Ihoriya, Tetsuya Yumoto, Noritomo Fujisaki, Kohei Tsukahara, Taihei Yamada, Yasuhiro Mandai, Takaaki Osako, Atsunori Nakao

**Affiliations:** Department of Emergency, Critical Care, and Disaster Medicine, Okayama University Graduate School of Medicine Dentistry and Pharmaceutical Sciences, Japan

## Abstract

Rectus sheath hematoma is an unusual but well-known clinical problem. Our hospital admitted a 54-year-old woman complaining of harsh right-sided hypogastric pain that started while muscle training. Computed tomography of the abdomen and pelvis demonstrated a right rectus sheath hematoma. As the hematoma did not increase, the patient was conservatively treated. Despite increased awareness of rectus sheath hematoma, its early diagnosis and treatment still present a challenge to emergency physicians. Swift acknowledgement of this rare cause of abdominal pain may avoid more intrusive examination, unnecessary hospitalization, and laparotomy. Careful consideration of the patient's medical history and a high index of suspicion are needed to diagnose this complication.

## 1. Introduction

Rectus sheath hematoma is a relatively uncommon but important cause of acute abdominal pain encountered in emergency room patients [1, 2]. Due to its clinical manifestation of acute abdominal pain with tenderness, it is often misdiagnosed and confused with other intra/extra abdominal problems, including abdominal wall abscess, abdominal wall tumors, hernias, and diverticular diseases, as well as gynecologic and urinary tract diseases, eventually leading to unnecessary laparotomy [3, 4].

Herein, we report a case of spontaneous rectus sheath hematoma following abdominal exercise. Rectus sheath hematoma is usually associated with abdominal trauma [4] and/or anticoagulation therapy [1, 5], as well as insulin injection [6], pregnancy [7, 8], abdominal surgery [9], exertion [10], and paroxysmal coughing [11]. However, our patient did not have any of these risk factors and may have experienced bleeding from the epigastric arteries and their branches in the rectus muscles during contraction of the rectus abdominis muscles. This case report aims to share with emergency physicians, basic treatment instructions for spontaneous rectus hematoma in terms of epidemiology, pathophysiology, predisposing factors, symptoms, and diagnosis. Physicians in surgery and primary/emergency medicine must become knowledgeable regarding this condition, as its misdiagnosis can lead to mortality, morbidity, and additional costs. [12]

## 2. Case Report

Our hospital admitted a 54-year-old woman complaining of strong, right-sided, hypogastric pain two hours after muscle training. The pain was exacerbated by breathing and moving. She was not taking anticoagulants and did not have any known blood dyscrasia. Her vital signs were pulse rate 80 beats/minute and rhythmic, blood pressure 115/85 mmHg, respiratory rate 18 breaths/minute, body temperature 37.8°C, and arterial oxygen saturation 97%. The patient had no symptoms of fever, nausea, chills, vomiting, or diarrhea. Physical examination revealed muscle defense and a tender, palpable 10 cm mass in the abdomen. Bruising around the umbilicus and flank was noted. Bowel sounds were normoactive. Testing revealed a white blood cell count of 11200 /mm^3^ (3500-9500 /mm^3^), hemoglobin 13.4 g/dL (12.1-15.1 g/dL), hematocrit 38% (37-46 %), and platelet count 365 x 10^3^ cells /mm^3^ (150-450 x 10^3^ cells /mm^3^). Her serum electrolyte, renal function, and urinalysis test results were not notable.

**Figure 1:**
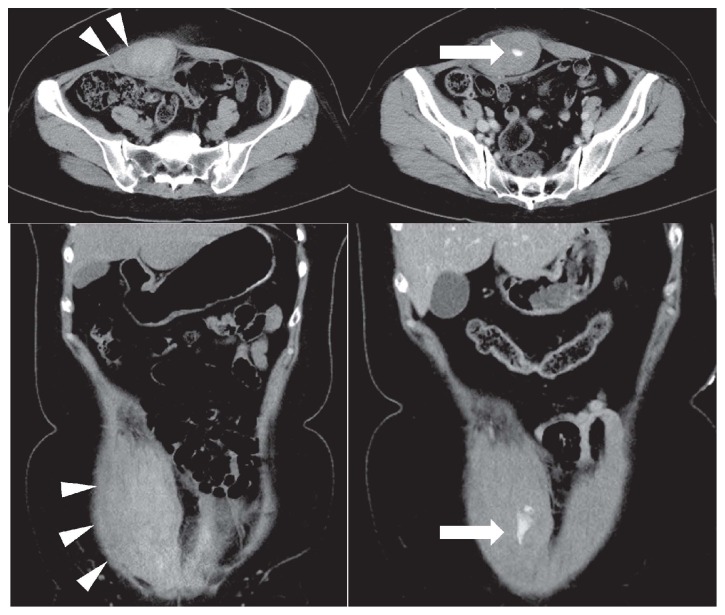
Abdominal and pelvic computed tomography demonstrated bilateral rectus sheath hematoma (white arrowhead). The hematoma extended into the median umbilical fold, forming a large mass that spread to the suprapubic area.

Abdominal computed tomography (CT) was performed to determine the reasons for acute abdomen, along with acute appendicitis. Enhanced abdominal CT revealed a right rectus sheath hematoma with extravasated contrasting agent (Figure 1). The hematoma extended downward into the lower abdominal wall and pelvis. Axial and sagittal CT images showed the rectus sheath hematoma with several 6 x 4 x 18 cm areas of active extravasation. Since her vital signs were stable, we started her on conservative therapy and discharged her four days after admission.

## 3. Discussion

One of the emergency physician's most important tasks is determining lethal causes of abdominal pain among cases mimicking a myriad of acute abdominal conditions such as rectus sheath hematoma. Rectus sheath hematoma may occur from not only a direct blow, but also strenuous non-contact exercise and trivial movements like twisting or sudden muscle strain and change in position without direct trauma. It occurs due to rupture of the upper and lower epigastric arteries and their branches or rupture of the rectus muscles themselves during contraction [13]. In our patient, we assume that the forceful contraction of the rectus muscles during exercise may have caused tearing of the epigastric vessel branches and led to rectus sheath bleeding.

Risk factors include anticoagulation therapy, old age, asthma exacerbation, recent surgery, medication injection in the abdomen, paroxysmal coughing, obesity, pregnancy, and chronic liver disease [11, 14, 15]. Our patient had no risk factors for bleeding except female gender. The period between the abdominal exercise and the patient's first complaints was about two hours. Regardless of this delay, exercise was likely the provoking event since no other physical trauma happened during this time. The patient's moderate bleeding intensity could help explain the delay.

With careful recording of the patient's medical history and paying close attention to a few critical points on examination, one could reliably differentiate a rectus hematoma from intra-abdominal pathology, thereby avoiding unnecessary surgery. Abdominal ultrasonography was also a very helpful available bedside tool and valuable screening technique in this case. Abdominal CT provides information helpful for differential diagnosis and can depict precise hematoma localization, with near 100% sensitivity during the first five days of rectus sheath hematoma formation [8]. In particular, accurately diagnosing the condition may be more challenging during pregnancy, as its presentation can mimic many common causes of abdominal pain specific to pregnancy. Management depends on hemorrhage severity, but in most cases, rectus sheath hematomas are self-limiting and are conservatively absorbed via rest and use of analgesics under close supervision. Any anticoagulant treatments should be terminated and when possible, replaced with coagulant factor or antidote. Surgery should be restricted to cases with large hematomas or free intra-abdominal ruptures [16]. When a patient is hemodynamically unstable, the hematoma size increases with a greater need for analgesics, or if the rupture has occurred in the peritoneum, consulting the surgery service is necessary.

## 4. Conclusion

Although uncommon, rectus sheath hematoma is a significant cause of abdominal pain that can imitate surgical acute abdomen. Physicians should consider this diagnosis in patients with the above described predisposing factors. It is important for emergency physicians to keep this differential diagnosis in mind along with other pathologies of acute abdomen.
